# Fabrication and Characterization of a Thermophone Based on Laser-Scribed Graphene Intercalated with Multiwalled Carbon Nanotubes

**DOI:** 10.3390/nano11112874

**Published:** 2021-10-28

**Authors:** Moin Rabbani, Aashir Waheed Syed, Syed Khalid, Mohammad Ali Mohammad

**Affiliations:** 1 School of Chemical and Materials Engineering (SCME), National University of Sciences and Technology (NUST), Sector H-12, Islamabad 44000, Pakistan; moinfuust@gmail.com (M.R.); syedaashirwaheed@gmail.com (A.W.S.); 2 Research Centre of Materials Science, Beijing Key Laboratory of Construction Tailorable Advanced Functional Materials and Green Applications, Beijing Institute of Technology, Beijing 100081, China; khalidsyedqau@yahoo.com

**Keywords:** thermophones, thermoacoustic, graphene oxide, laser-scribed graphene, multiwalled carbon nanotubes, pulse width modulation, sound pressure level

## Abstract

The low sound pressure level and high operating voltages of thermophones have limited their applications in the past. However, in recent years, utilizing nanomaterials in thermophones has improved their efficiency and applicability. Nanomaterials, especially carbon nanotubes and graphene, have the advantage of low heat capacity per unit area (HCPUA) and high electrical and thermal conductivity. Therefore, they require a low electrical input power and generate a high sound pressure level (SPL) by efficiently transferring heat to the surrounding fluid. Laser-scribed graphene (LSG) can generate smooth spectra acoustic emissions over a wide range of frequencies by means of thermoacoustic (TA) emission. In this work, a thermophone based on LSG intercalated with multiwalled carbon nanotubes (MWCNTs) is proposed. The effects of varying input power, duty cycle percentage and measuring distance on the sound pressure level (SPL) of thermophones are studied to extract maximum efficiency. The achieved SPL of LSG, normalized to the input power, has increased by approximately 11 dB by intercalating it with MWCNTs, which shows that our proposed material can be a potential candidate for an efficient thermophone.

## 1. Introduction

In conventional loudspeakers, sound is produced by the mechanical vibration of a diaphragm while in thermoacoustic loudspeakers, sound is produced by exchanging the produced Joule heating to the surrounding fluid and generating acoustic waves, without the need of any physically vibrating parts. Thermophones are lightweight, flexible, transparent and low cost, which makes them a better alternative to conventional loudspeakers. Thermophones do not require any mechanical parts and rare earth metals, which also makes them preferable over conventional loudspeakers.

Thermophones, or thermoacoustic loudspeakers, were first invented by Arnold and Crandall in 1917 [1]. Thermoacoustic loudspeakers can be fabricated on large, flexible substrates and have a wide frequency response and lower fluctuations [2]. Due to the non-harmonic nature of thermoacoustic sound generators, they are precision sources of sound [3]. The thermophone made by Arnold and Crandall consisted of a thin platinum wire. The progress in thermophones halted for a century due to the high heat capacity of bulk materials, which caused the low yield of thermophones at high operating voltages. However, in 1999, Shinoda et al. fabricated a thermophone based on 30 nm thin aluminum film on porous silicon, which led to a significant improvement in its performance due to the low heat capacity of nano-level thin film [4]. Since then, many groups have been working on improving the performance of thermoacoustic loudspeakers by utilizing nanotechnology.

In 2008, Xiao et al. fabricated a flexible carbon-nanotube (CNT) thin-film thermophone. The CNT’s thin films have the advantage of being flexible, stretchable and transparent [2]. Moreover, the thin films of CNTs can be tailored into many shapes and sizes and can be coated on a variety of flexible substrates [2]. The SPL of a 9 cm^2^ CNT film was 88 dB at 1 W input power, 20 kHz frequency and a 5 cm measuring distance (distance from loudspeaker to microphone). In 2014, Fei et al. fabricated low-voltage-driven graphene-foam earphones by growing graphene on nickel foam and then etching nickel [5]. The graphene obtained by this method was highly porous with a low heat capacity per unit area (HCPUA) and a high surface area 3D structure, which increased the SPL of the thermophone. The SPL of a graphene-foam loudspeaker of 1 cm^2^ area was 75 dB at 1 W input power, 10 kHz frequency and a measuring distance of 3 cm. The SPL was enhanced considerably, but the process was costly, time consuming and not scalable. In 2011, Tian et al. fabricated loudspeakers based on silver nanowire films using glass and PET as a substrate [6]. The silver-nanowire thermophone was highly transparent, but its fabrication process was based on a dry-transfer technique, which is expensive and time consuming. In 2014, Dutta et al. fabricated a gold-nanowire thin-film thermophone with SPL of 41 dB at 0.6 W input power, 10 kHz frequency and 3 cm measuring distance [7]. The efficiency of the gold-nanowire thermophone was very low, and the fabrication process used was a lithography-patterned nanowire electrodeposition method, which is very costly and time consuming. In 2015, Tian et al. compared the SPL of 1 to 6 layers graphene [8]. The SPL decreased with the increasing number of layers due to the fact that increasing the number of stacked graphene layers causes an increase in HCPUA.

In 2019, Romanov et al. fabricated a thermophone based on freestanding single-walled carbon nanotubes (SWCNTs) and studied the effect of film thickness and purity on sound pressure level (SPL) [9]. The thin films of the SWCNTs were prepared by a chemical vapor deposition (CVD) process and purified under vacuum conditions by annealing at a temperature greater than 1200 °C. The SPL was improved with purification of the SWCNTs, but the purification process was time consuming and needed vacuum and high temperature requirements. In 2019, Huang et al. studied the effect of power, thickness of graphene film, substrate (paper, Si and PMMA) and distance on SPL [10]. The graphene film with a thickness of 20 nm, fabricated on paper substrate, generated the highest SPL. In 2020, the issue of film breakage in the thermophone was addressed by Kang et al. who fabricated a thermophone by utilizing a self-healing polymer [11]. These thermophones were based on AgNWs (silver nanowires) and PUHUA (poly urethane-hindered urea) composite electrodes that can be healed after film breakage by heating at 90 °C and 80% humidity. In 2020, Romanov et al. utilized Joule heating for the purification of CVD-grown SWCNTs. The purity of the films increased by increasing the temperature. The sound pressure level was enhanced by film purification, but the method was costly and time consuming due to vacuum requirements [12]. All the above-mentioned methods for enhancing the SPL are time consuming, costly and often require high temperatures and vacuum conditions. Therefore, simple and low-cost alternatives for enhancing the SPL are needed for the scalable production of thermophones, and the need of a simple, one-step thermophone-fabrication method is inevitable.

Laser scribing is a simple, one-step method for the fabrication of electrodes for different applications. In 2017, Tao et al. fabricated a thermophone by a simple fabrication method of laser scribing a polyimide (PI) sheet [13]. The fabrication process consisted of one step, and the cost of the process was also very low. However, a high laser power was used to reduce the polyimide sheet, which can deform the substrate. An SPL of 53 dB was achieved from a 2 cm^2^ thermophone at an input power of 0.42 W, 20 kHz frequency and 2.5 cm measuring distance. In 2014, Tian et al. exploited laser-scribing technology to fabricate a graphene earphone by using laser-scribed graphene [14]. An SPL of 35 dB was achieved at 1 W input power and 10 kHz frequency from a 1 cm^2^ area thermophone. The main advantage of using LSG as a thermophone is that air gaps exist between the graphene layers, which supports the flow of thermal energy to the fluid and prevents the flow of thermal energy to the substrate [14]. Sound generation by LSG has been extensively studied by varying various parameters like laser power, scanning speed, measuring distance and substrate thickness [15,16]. However, the study of the SPL of LSG by varying the number of layers, air gap and surface area has never been done before.

In this work, the effect of decreasing the number of layers and increasing the air gap between them on the SPL of LSG-based thermophones is discussed. Two square-shaped thermophones of 1 × 1 cm^2^ dimensions are fabricated based on LSG and LSG intercalated by MWCNTs, respectively. The sound pressure level of both thermophones is compared. The effects of measuring distance, duty cycle percentage and input voltage on SPL of both thermophones are also discussed in this work.

## 2. Material and Methods

### 2.1. Material Synthesis

**Figure 1:**
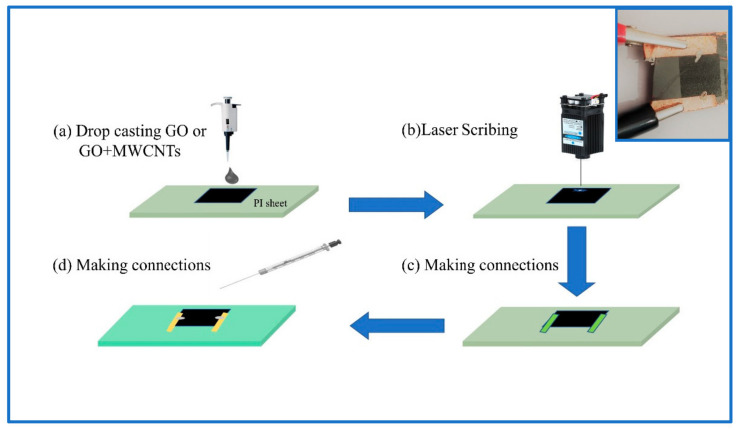
Schematic of device fabrication, (**a**) drop casting of GO and GO+MWCNTs solution on polyimide substrate, (**b**) laser scribing the dried GO and GO+MWCNTs membranes, (**c**,**d**) applying connections by using copper tape and silver paste. Inset shows the picture of the LSG+MWCNTs-based thermophone.

Graphene oxide (GO) was prepared by the modified Hummer’s method [17]. Multiwalled carbon nanotubes were purchased from Sigma-Aldrich (St. louis, MO, USA). A 3 mg mL^−1^ GO solution was prepared by mixing 60 mg GO powder in 20 mL distilled water and sonicating for 2 h. The MWCNTs were first cleaned by ultrasonicating them for five hours in a 3:1 solution of sulfuric acid and nitric acid. The impurity-free MWCNTs were then collected by centrifuging and filtration. The filtered MWCNTs were then dried in a vacuum oven at a temperature of 60 °C for 12 h. A solution was then prepared by mixing 6 mg of MWCNTs and 60 mg of GO in 20 mL of deionized water. The solution was then sonicated for 5 h by means of a probe sonicator (KS500F, Shanghai, China) at an interval of 5 s, which resulted in the induced insertion of MWCNTs between the layers of GO [18]. This hybrid solution with GO intercalated by the MWCNTs is named as GO+MWCNTs. Next, 0.1 mL of both GO and GO+MWCNTs hybrid solutions were drop casted separately onto the two 0.15 mm thick PI sheets of 2 × 3 cm^2^ area and dried at ambient temperature. The prepared membrane of GO and GO+MWCNTs was then laser scribed using a 450 nm pulsed laser into a square pattern of 1 × 1 cm^2^ area by applying the same laser power. After laser scribing, the products are named laser-scribed graphene (LSG) and the LSG and MWCNTs hybrid (LSG+MWCNTs), respectively. Then, copper tape and silver paste were applied to make connections on both devices. Figure 1 shows a schematic of this process. We named the LSG-based device as thermophone-1 (TP-1) and the LSG+MWCNTs-based device as thermophone-2 (TP-2).

### 2.2. Structural and Electrical Characterization

The devices were characterized by their material properties as well as their acoustic properties. The material properties were characterized using the JEOL Analytical SEM (JSM-6490A, Tokyo, Japan) with 3 nm resolution and 2D non-contact profilometer (PS-50, NANOVEA, USA) and a Raman spectrometer (BWS415-532s-iRaman, Newark, NJ, USA) with 532 nm laser excitation. This Raman spectrometer covers a range from 59 to 4000 cm^−1^ with a spectral resolution of less than 4.5 cm^−1^ at 614 nm. The electrical characterization was performed by using an electrochemical workstation (BioLogic, Science Instruments, SP-50, USA) in the two-probe mode to determine I − V characteristics.

### 2.3. Acoustic Characterization

**Figure 2:**
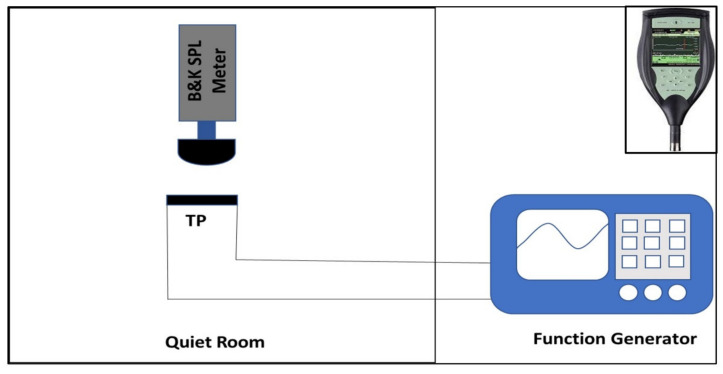
Schematic of sound testing setup. Inset shows B&K 2250 SPL meter.

Acoustic measurements were taken in a quiet room with a background SPL of 30 dB. A function generator (Twintex TFG-3205E, Shenzhen, China) was used for supplying electrical signals to thermophones, and also a SPL meter (B&K Type 2250, Naerum, Denmark) was used for measuring the SPL of the sound generated by the thermophones. A 5 V AC signal was employed to drive the thermophone. To suppress the frequency doubling effect, a DC signal of same magnitude was superimposed on the AC signal. The frequency of the input signal was varied from 0 Hz to 20 kHz, and the acoustic response of the thermophones was measured. The distance from the SPL meter to the thermophones was varied from 1 cm to 6 cm to measure their SPL response by using a 5 V AC + 5 V DC signal with 10 kHz frequency. A pulse width modulation (PWM) signal of 10 V and 10 kHz frequency was also used to drive a thermophone to measure its acoustic response upon various duty cycles. Furthermore, the effect of increasing voltage on the SPL was also measured using PWM signals (50% duty cycle) of different voltages with a 10 kHz frequency and a measuring distance of 1 cm. Figure 2 shows the schematic of this testing setup, and the inset shows the B&K 2250 SPL meter.

## 3. Results and Discussion

### 3.1. Structural Characterization

**Figure 3:**
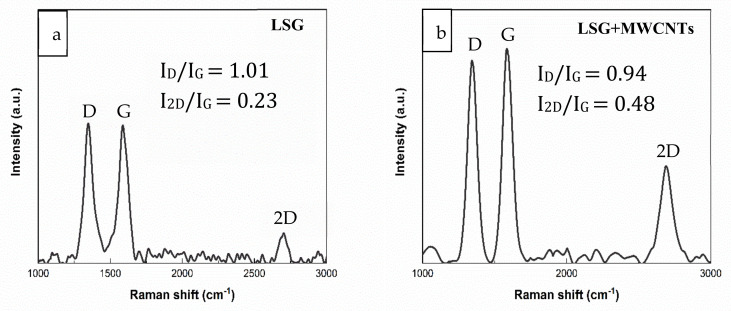
(**a**) Raman spectra of LSG, (**b**) Raman spectra of LSG+MWCNTs.

Material characterization was performed via Raman spectroscopy, SEM imaging and optical profilometry. Raman spectra of LSG and LSG+MWCNTs are shown in Figure 3a,b, respectively.

**Figure 4:**
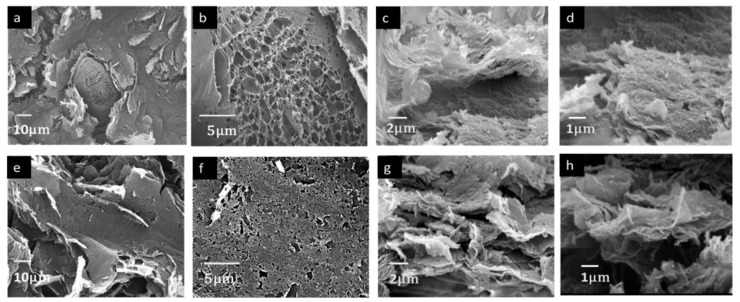
(**a**,**b**) SEM Images of LSG, (**c**,**d**) cross-section view of LSG, (**e**,**f**) SEM Images of LSG+MWCNTs, (**g**,**h**) Cross-section view of LSG+MWCNTs.

The peaks of LSG at 1342 cm^−1^ and 1599 cm^−1^ corresponded to sp^3^ (D band) and sp^2^ (G band), respectively. Similarly, the peaks at 1345 cm^−1^ and 1587 cm^−1^ for LSG+MWCNTs corresponds to the D band and G band, respectively, in Figure 3b. The G band is common to all carbon-based materials while the D band shows the degree of crystallinity in the carbon structure. The intensity ratio of the two peaks (I_D_/I_G_) is used to measure the degree of disorder in the material [19]. The I_D_/I_G_ of LSG was found to be 1.01, which is higher than the I_D_/I_G_ of LSG+MWCNTs (0.94); this is mainly due to decreased crystallinity of LSG. The increase in the crystallinity of LSG+MWCNTs is due to the presence of crystalline MWCNTs. The Raman spectra of GO shows the absence of a 2D peak, as shown in Figure S1. The 2D peak in LSG is observed at 2700 cm^−1^ and in LSG+MWCNTs at 2690 cm^−1^. The peak shift in the Raman spectra of graphene is related to the change in the electronic and structural properties [20]. The 2D peak for carbon-based materials indicates the presence of graphene. The ratio I_2D_/I_G_ is used to compare the number of graphene layers [21]. I_2D_/I_G_ is inversely proportional to the number of graphene layers. The I_2D_/I_G_ of LSG is 0.23, which shows the presence of stacked graphene layers or graphene flakes. The I_2D_/I_G_ of LSG+MWCNTs is increased to 0.48, identical to a few layers of graphene, which shows the decrease in the number of layers of graphene. The decrease in the number of layers of LSG+MWCNTs is due to the insertion of MWCNTs between the layers of graphene. SEM Images of LSG and LSG+MWCNTs are shown in Figure 4.

Figure 4a,b shows the SEM Images of LSG at different magnifications. Flakes of graphene with a foam-like structure can be observed in LSG. Figure 4c,d shows the cross-section view of LSG at different magnifications. Thick graphene flakes can be observed in the cross-section view of LSG. Figure 4e,f shows the SEM Images of LSG+MWCNTs. A foam-like structure with a high porosity in comparison to that of LSG is observed in the SEM Images of LSG+MWCNTs. The cross-section view of LSG+MWCNTs is shown in Figure 4g,h. By comparing the cross-section view of LSG and LSG+MWCNTs, it can be observed that more of an air gap exists between the sheets of LSG+MWCNTs. The thickness of the graphene flakes is also less in LSG+MWCNTs. The uniform structure of LSG and LSG+MWCNTs can be observed in Figure S2.

Film thickness is measured by optical profilometry. The maximum thickness of GO is 16.9 µm (Figure S3) while the maximum thickness of GO+MWCNTs is 23.9 µm (Figure S4). The maximum thickness of GO+MWCNTs is therefore higher than that of GO. This could be the result of an increase in hydrophobicity due to the presence of MWCNTs in the GO+MWCNTs solution, which resulted in the formation of a thick layer upon drying. The maximum thickness of LSG is 112 µm (Figure S5), and the maximum thickness of LSG+MWCNTs is 166 µm (Figure S6). Upon laser ablation, the gases evaporate from the material, which creates a local pressure. This local pressure causes the sheets of graphene to expand, which then increase the thickness of material. The thickness of LSG+MWCNTs is greater than LSG due to the presence of MWCNTs between the layers of LSG. The MWCNTs prevent the restacking of LSG layers after laser ablation [18].

**Figure 5:**
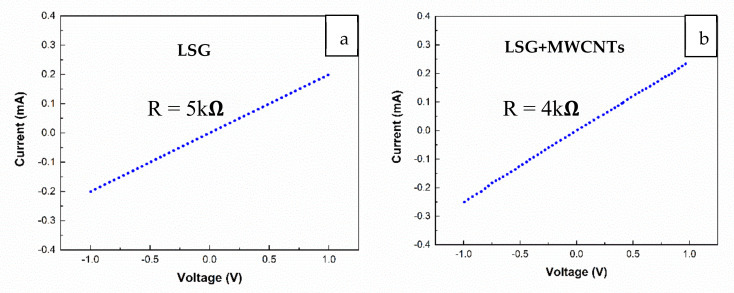
(**a**) I − V characteristics of LSG, (**b**) I − V characteristics of LSG+MWCNTs.

The I − V curve of LSG and LSG+MWCNTs is shown in Figure 5a,b, respectively. The resistances of LSG and LSG+MWCNTs are 5 kΩ and 4 kΩ, respectively, which shows that the electrical conductivity of LSG+MWCNTs increases due to the presence of MWCNTs.

The temperature profile of LSG and LSG+MWCNTs is measured by using a thermal imaging camera (FLIR T530, Wilsonville, Oregon, USA). Figure S7a shows the temperature profile of TP-1 at 0 V DC, which confirms the absence of any thermal activity when thermophones are not attached to any power source. Figure S7b shows the temperature profile of TP-1 at 9 V DC, which confirms the generation of thermal activity. Figure S7c shows the temperature profile of TP-2 at 9 V DC. An increase in thermal activity, or temperature, is observed in TP-2 as compared to TP-1 at the same voltage. This thermal activity confirms the presence of a Joule heating effect in both LSG and LSG+MWCNT-based thermophones.

### 3.2. Acoustic Characterization

**Figure 6:**
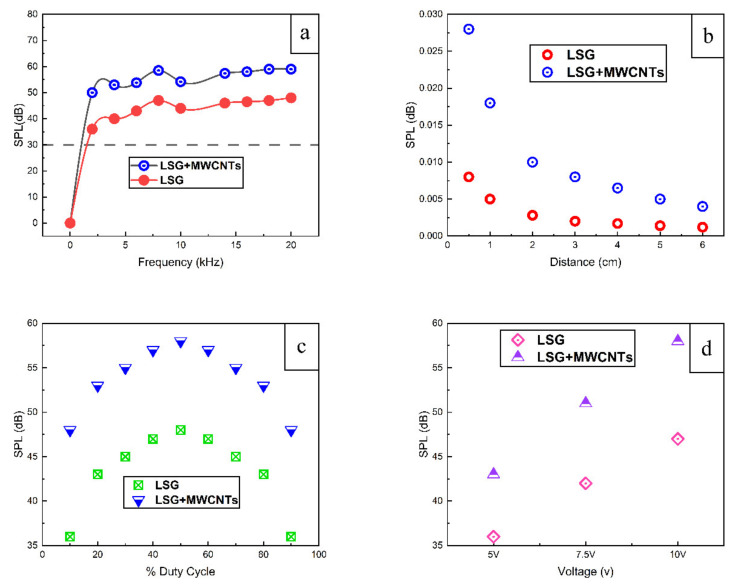
Comparison of SPL of TP-1 and TP-2, (**a**) by varying frequency, (**b**) by varying measuring distance, (**c**) by varying % of duty cycle, and (**d**) by varying input voltage.

The sound performance of both thermophones is tested by applying an AC signal of 5 V with a 5 V DC offset. The frequency is swept from 0 Hz to 20 kHz. A comparison of the SPL of TP-1 and TP-2 is shown in Figure 6a.

When an input electrical power is applied to the thermophone, thermal energy is generated within it. Thermal energy is divided into three parts: (1) The thermal energy consumed within thermophone, (2) the thermal energy flowing towards the substrate and (3) the thermal energy flowing towards the fluid/air.

It is the thermal energy that flows towards air that contributes to sound generation. Therefore, when making an efficient thermophone, the flow of energy towards the substrate and the consumption of thermal energy within the thermophone material should be minimized in order to achieve the maximum flow of energy towards the fluid/air.

Asadzadeh et al. established the relation between fluid thermal energy and sound pressure level [22] as

(1) $$P_{\mathrm{rms}}=\frac{m_{\mathrm{air}}\cdot \;f}{\surd 2C_{p}T_{o}r}\;\times \;{\overset{\cdot }{Q}}_{\mathrm{fluid}}$$

where, *P_rms_* is the mean sound pressure level, *m_air_* is the molecular mass of air, *f* is the input frequency of the sound source, $C_{p}$ is the heat capacity at a constant pressure of air, $T_{o}$ is the temperature of the thermophone, r is the measuring distance between thermophone and microphone and $\dot{Q}$ is the fluid thermal energy.

The fluid thermal energy is directly proportional to the SPL of the thermophone. Therefore, the SPL can be enhanced by increasing the fluid thermal energy. There are plenty of parameters, such as HCPUA, surface area and the thermal conductivity of the thermophone material, that can be manipulated in order to increase the fluid thermal energy.

From Figure 6a, we can see that the maximum SPL produced by TP-2 is 11 dB higher than that of TP-1. At least 5 samples were prepared for both LSG and LSG+MWCNTs. The SPL variation of ±1.5 dB was observed upon measurement. The samples with an average SPL are plotted in Figure 6a. The induced insertion of MWCNTs between the layers of LSG prevents the restacking and agglomeration of LSG layers and hence improves the air-accessible surface area. More air molecules can interact with the heat-generating surface, which causes an efficient conduction of heat into air and prevents leakage of heat to the substrate. Moreover, the HCPUA of agglomerated or stacked LSG is greater than the intercalated LSG [8]. In theoretical terms, the low HCPUA of the thermophone material minimizes the consumption of thermal energy within the thermophone material itself, which causes the efficient flow of energy towards the fluid. Therefore, the SPL of TP-2 is greater than that of TP-1. CNTs have a very high thermal conductivity, which prevents the flow of heat towards the substrate and facilitates the flow of heat towards the air. Moreover, CNTs also have thermoacoustic properties [2]. Due to the high surface area, low HCPUA, high thermal conductivity of MWCNTs and the synergetic effect of sound emission from LSG and MWCNTs in the LSG+MWCNTs, the SPL produced by the TP-2 is higher than that of TP-1.

From Equation (1), it can be seen that there is an inverse relation between the SPL and the distance between the thermophone and the sound measuring instrument. Figure 6b shows the effect of changing the measuring distance on SPL. It can be observed that the SPL of both TP-1 and TP-2 decreased by increasing measuring distance, which supports the theory.

The SPL of TP-1 and TP-2 is measured by changing the % duty cycle of PWM. From Figure 6c, it can be seen that by increasing the % duty cycle up to 50%, the sound pressure level is increased. At 50% duty cycle, both thermophones produced their highest SPL. By further increasing the % duty cycle, SPL decreased. Figure 6d shows the effect of varying input voltage on output SPL. The SPL of both thermophones increased upon increasing the input voltage of the PWM signal.

From Table 1, it can be shown that the SPL of TP-2 is considerably higher than previously reported for LSG-based thermophones.

## 4. Conclusions

In summary, we proposed an efficient thermophone material by intercalating laser-scribed graphene using multiwalled carbon nanotubes. The insertion of multiwalled carbon nanotubes between the layers of laser-scribed graphene caused an enhancement in the sound pressure level by 11–24 dB as compared to using LSG only. This improvement is due to a decrease in the number of layers of laser-scribed graphene and an increase in the thermal conductivity and surface area of the material. Furthermore, the effect of measuring distance, input voltage and duty cycle percentage are also discussed for harvesting the best performance from this novel thermophone.

## Figures and Tables

**Table 1:** Comparison of this work with published results.

Material	Excitation Conditions	Measuring Distance	SPL (dB)	References
LSG	5 V AC+5 V DC, 1 cm^2^	1 cm	35	*ACS Nano* 2014 [14]
LSG	1 W, 1 cm^2^	3 cm	40	*Journal of Applied Physics* 2015 [23]
LSG	5 V AC + 5 V DC, 1 cm^2^	1 cm	40	2014 IEEE 27th International Conference on Micro Electro Mechanical Systems [24]
LSG	5 V AC + 5 V DC, 1 cm^2^	1 cm	48	This work
LSG+MWCNTs	5 V AC + 5 V DC, 1 cm^2^	1 cm	59	This work

## Data Availability

Data is contained within the article or Supplementary Information.

## Supplementary Materials

The following are available online at https://www.mdpi.com/article/10.3390/nano11112874/s1, Figure S1: Raman spectra of graphene oxide, Figure S2: (a) SEM image of GO, (b) SEM image of GO+MWCNTs, (c) SEM image of LSG, (d) SEM image of LSG+MWCNTs, Figure S3: (a) Height profile of GO, (b) Intensity profile of GO, (c) Thickness profile of GO, Figure S4: (a) Height profile of GO+MWCNTS, (b) Intensity profile of GO+MWCNTs, (c) Thickness profile of GO+MWCNTs, Figure S5: (a) Height profile of LSG, (b) Intensity profile pf LSG, (c) Thickness profile of LSG, Figure S6: (a) Height profile of LSG+MWCNTs, (b) Intensity profile of LSG+MWCNTs, (c) Thickness profile of LSG+MWCNTs. Figure S7: Thermal images of thermophones by applying (a) 0V to LSG based thermophone, (b) 9VDC to LSG based thermophone, (c) 9V DC to LSG+MWCNTs based thermophones.
