# Supplementary material for: Fabrication and Characterization of a Thermophone Based on Laser-Scribed Graphene Intercalated with Multiwalled Carbon Nanotubes
Source: Nanomaterials (Basel). 2021 Oct 28;11(11):2874. doi: 10.3390/nano11112874 (PMC8621151; doi:10.3390/nano11112874)
Supplement: Supplementary file 1 [file nanomaterials-11-02874-s001.zip › nanomaterials-1321493-supplementary.pdf]

Supplementary Materials

Fabrication and Characterization of a Thermophone Based on Laser-Scribed Graphene Intercalated with Multiwalled Carbon Nanotubes

Moin Rabbani [1], Aashir Waheed Syed [1], Syed Khalid [2] and Mohammad Ali Mohammad [1,*]

[1] School of Chemical and Materials Engineering (SCME), National University of Sciences and Technology (NUST), Sector H-12, Islamabad 44000, Pakistan; moinfuust@gmail.com (M.R.); syedaashirwaheed@gmail.com (A.W.S); dr.ali@nust.edu.pk (M.A.M)

[2] Research Centre of Materials Science, Beijing Key Laboratory of Construction Tailorable Advanced Functional Materials and Green Applications, Beijing Institute of Technology, Beijing 100081, China; khalidsyedqau@yahoo.com (S.K.)

* Correspondence: dr.ali@nust.edu.pk

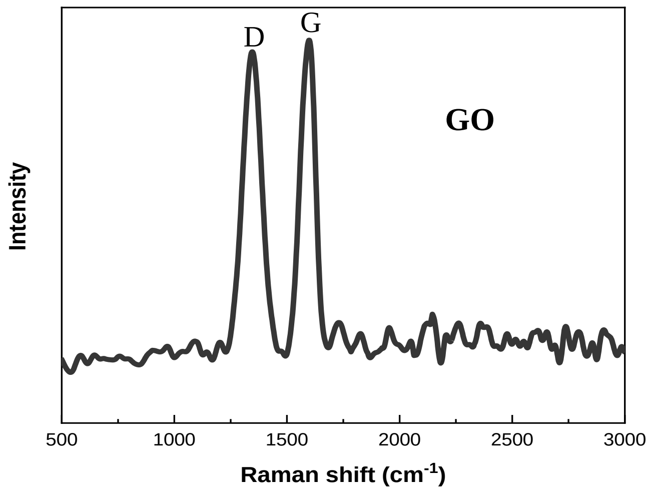

Figure S1. Raman spectra of graphene oxide

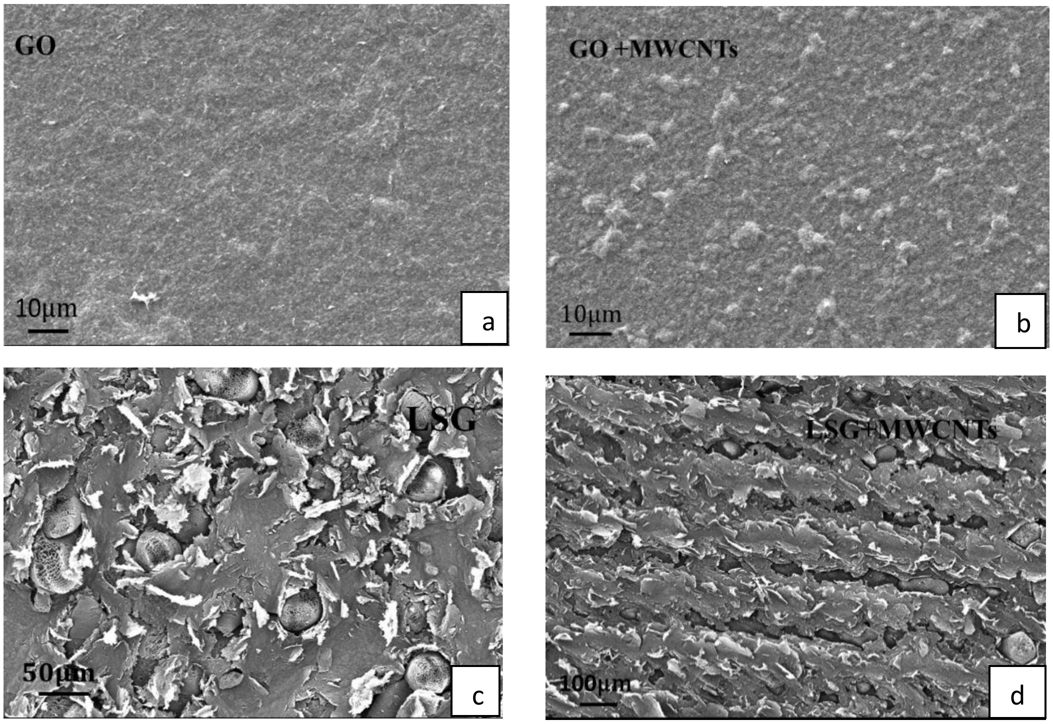

Figure S2. (**a**) SEM image of GO, (**b**) SEM image of GO+MWCNTs, (**c**) SEM image of LSG, (**d**) SEM image of LSG+MWCNTs

Optical Profilometry of GO

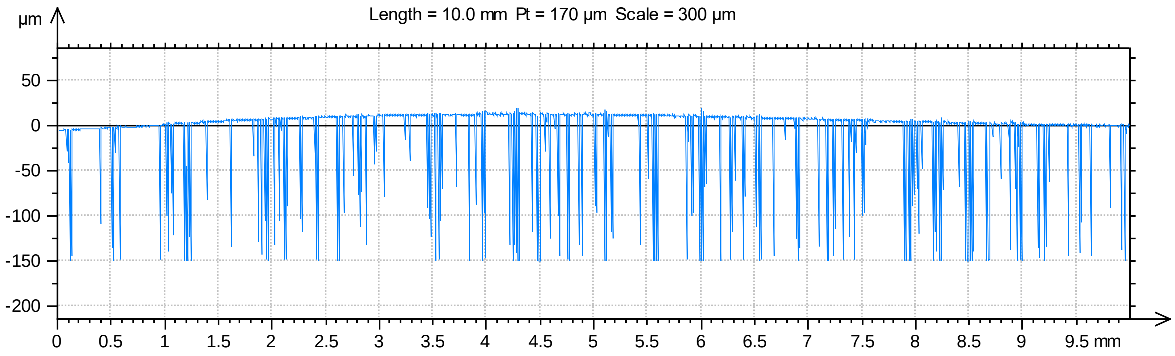

Figure S3. (**a**): Height profile of GO

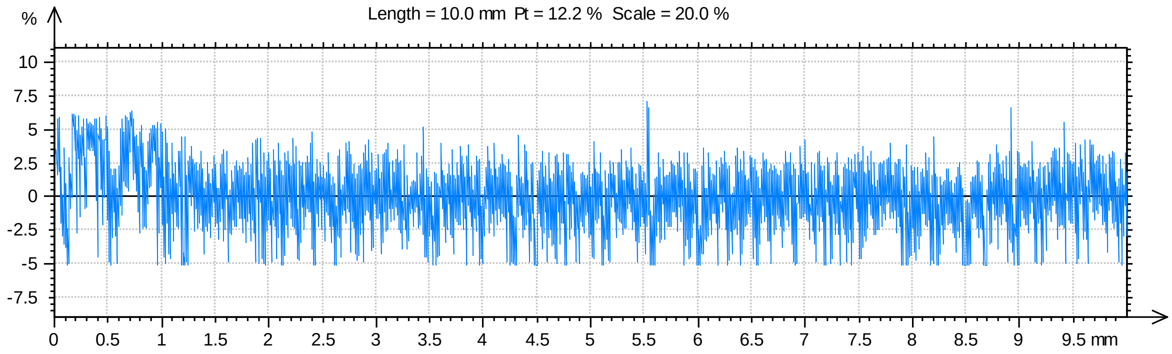

Figure S3. (**b**): Intensity profile of GO

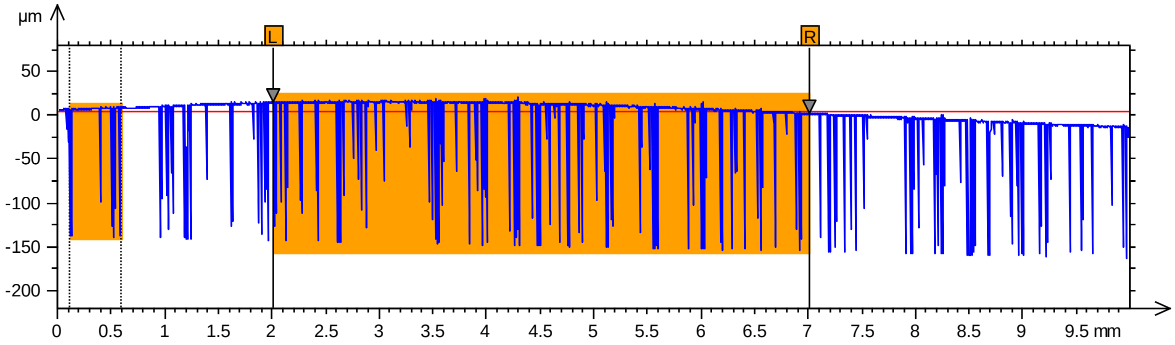

	1
Maximum height	16.9 µm
Mean height	1.23 µm
Width	5.00 mm

Figure S3. (**c**): Thickness profile of GO

Optical profilometry of GO+MWCNTs

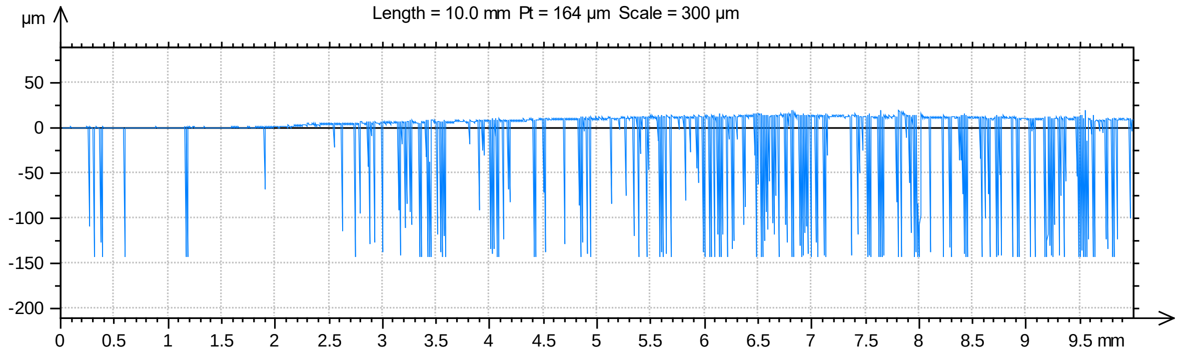

Figure S4. (**a**): Height profile of GO+MWCNTs

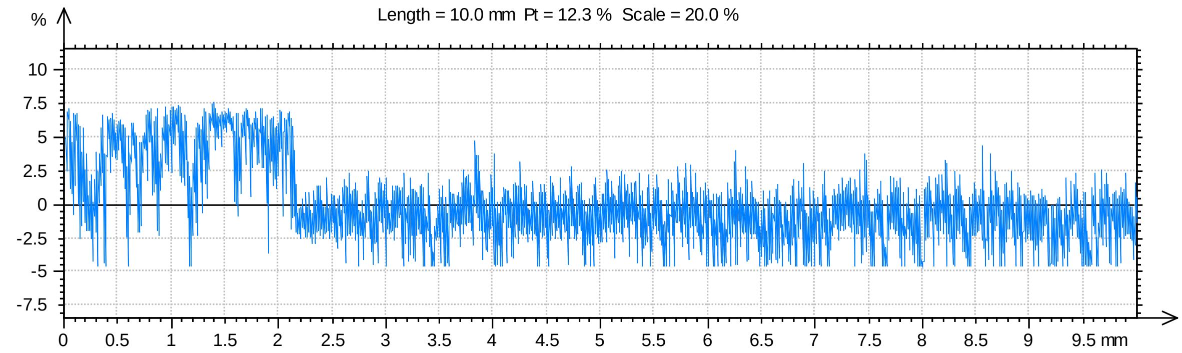

Figure S4. (**b**): Intensity profile of GO+MWCNTs

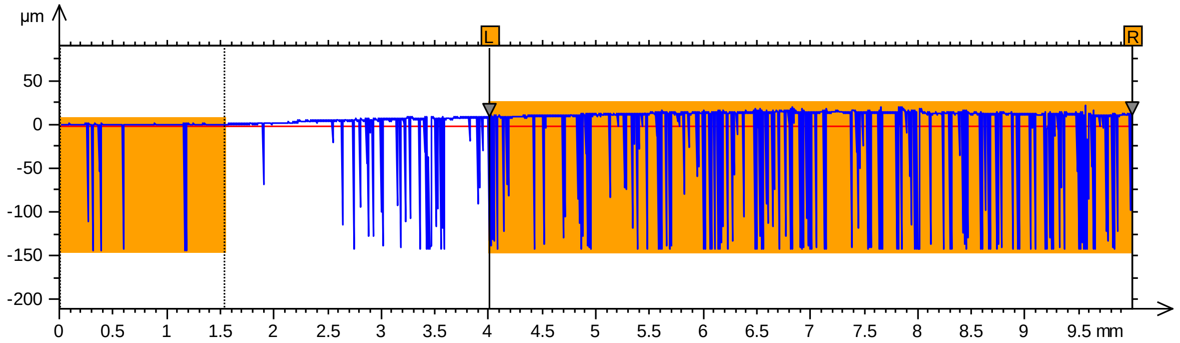

	1
Maximum height	23.9 µm
Mean height	1.32 µm
Width	6.00 mm

Figure S4. (**c**): Thickness profile of GO+MWCNTs

Optical profilometry of laser scribed graphene (LSG)

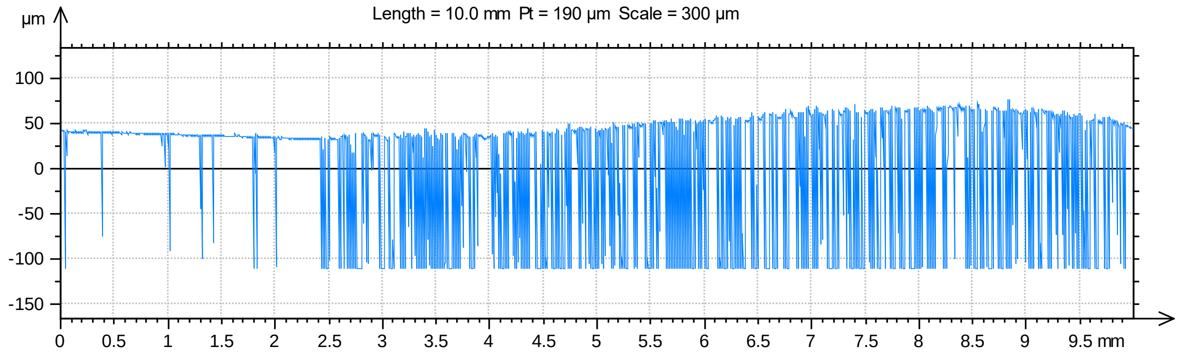

Figure S5. (**a**): Height profile of LSG

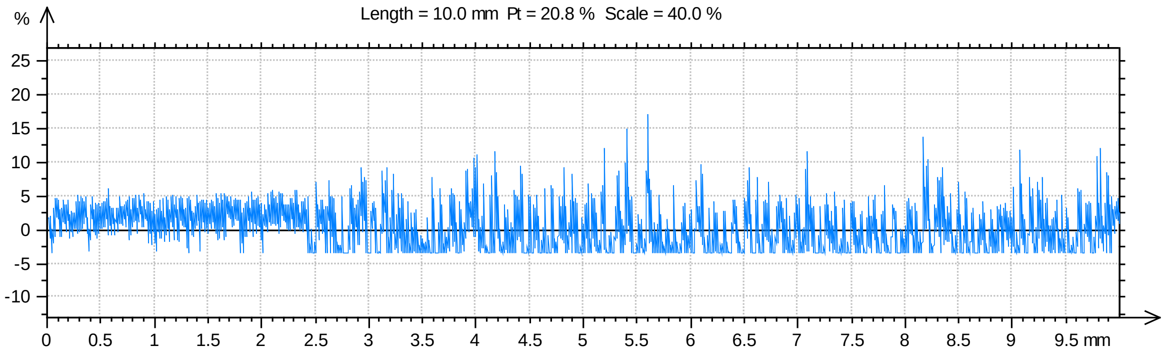

Figure S5. (**b**): Intensity profile of LSG

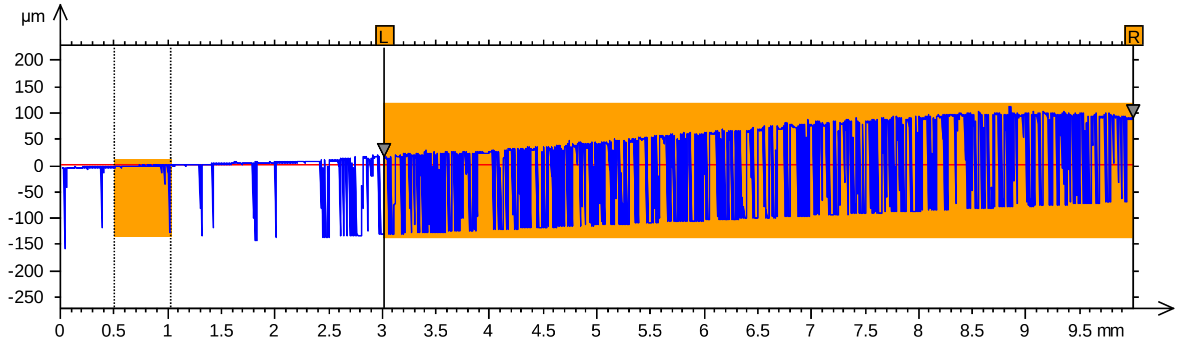

	1
Maximum height	112 µm
Mean height	2.09 µm
Width	6.98 mm

Figure S5. (**c**): Thickness profile of LSG

Optical profilometry of multiwalled carbon nanotubes intercalated laser scribed graphene (LSG+MWCNTs)

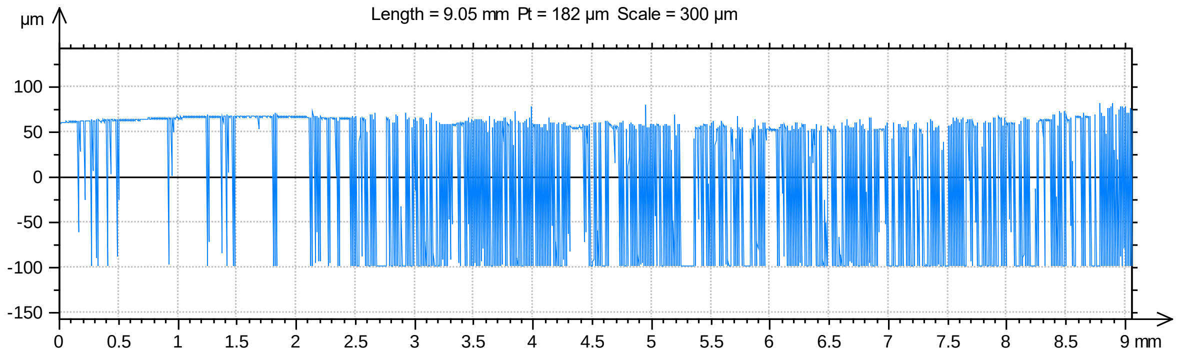

Figure S6. (**a**): Height profile of LSG+MWCNTs

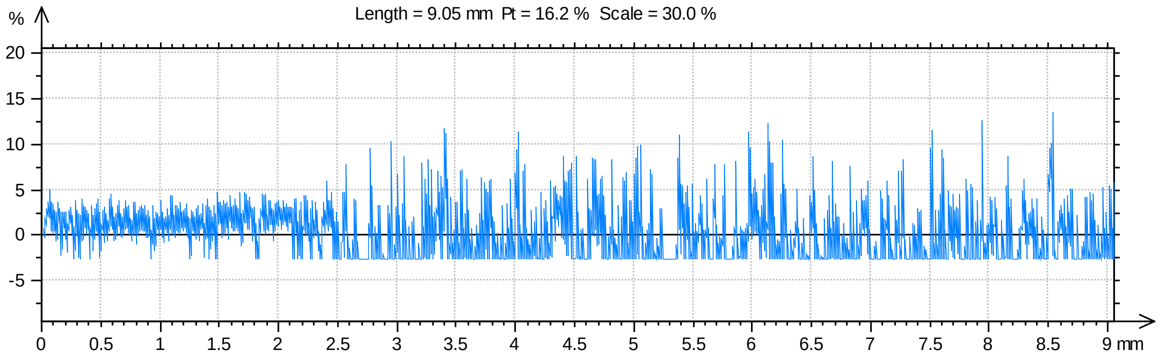

Figure S6. (**b**): Intensity profile of LSG+MWCNTs

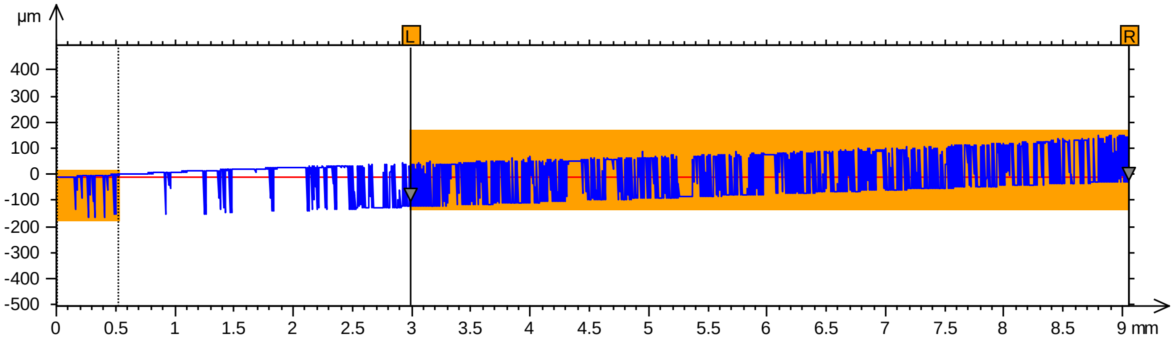

	1
Maximum height	166 µm
Mean height	15.1 µm
Width	6.07 mm

Figure S6. (**c**): Thickness profile of LSG+MWCNTs

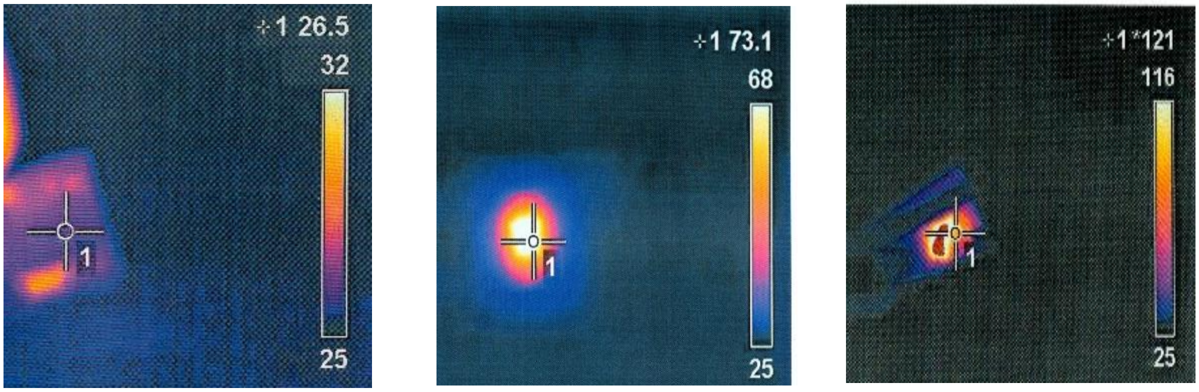

Figure S7. Thermal images of thermophones by applying (**a**) 0V to LSG based thermophone, (**b**) 9V DC to LSG based thermophone, (**c**) 9V DC to LSG+MWCNTs based thermophone
